# Radiomics Analysis Based on Automatic Image Segmentation of DCE-MRI for Predicting Triple-Negative and Nontriple-Negative Breast Cancer

**DOI:** 10.1155/2021/2140465

**Published:** 2021-08-10

**Authors:** Mingming Ma, Liangyu Gan, Yuan Jiang, Naishan Qin, Changxin Li, Yaofeng Zhang, Xiaoying Wang

**Affiliations:** ^1^Department of Radiology, Peking University First Hospital, Beijing, China; ^2^Breast Disease Center, Peking University First Hospital, Beijing, China; ^3^Beijing Smart Tree Medical Technology co. Ltd., Beijing, China

## Abstract

**Purpose:**

To investigate whether quantitative radiomics features extracted from dynamic contrast-enhanced magnetic resonance imaging (DCE-MRI) could be used to differentiate triple-negative breast cancer (TNBC) and nontriple-negative breast cancer (non-TNBC).

**Materials and Methods:**

This retrospective study included DCE-MRI images of 81 breast cancer patients (44 TNBC and 37 non-TNBC) from August 2018 to October 2019. The MR scans were achieved at a 1.5 T MR scanner. For each patient, the largest tumor mass was selected to analyze. Three-dimensional (3D) images of the regions of interest (ROIs) were automatically segmented on the third DCE phase by a deep learning segmentation model; then, the ROIs were checked and revised by 2 radiologists. DCE-MRI radiomics features were extracted from the 3D tumor volume. The patients were randomly divided into training (*N* = 57) and test (*N* = 24) cohorts. The machine learning classifier was built in the training dataset, and 5-fold cross-validation was performed on the training cohort to train and validate. The data of the test cohort were used to investigate the predictive power of the radiomics model in predicting TNBC and non-TNBC. The performance of the model was evaluated by the area under receiver operating characteristic curve (AUC), accuracy, sensitivity, and specificity.

**Results:**

The radiomics model based on 15 features got the best performance. The AUC achieved 0.741 for the cross-validation, and 0.867 for the independent testing cohort.

**Conclusion:**

The radiomics model based on automatic image segmentation of DCE-MRI can be used to distinguish TNBC and non-TNBC.

## 1. Introduction

Breast cancer is a heterogeneous disease with different clinical behavior, subtypes, and treatment responses [1, 2]. There are four main intrinsic molecular subtypes of breast cancer: luminal A, luminal B, human epidermal growth factor receptor 2- (HER2-) enriched, and triple-negative [3]. Assessment of molecular subtypes is currently based on either gene expression profiling or immuno-histochemical (IHC) [4], which all require invasive tumor sampling. However, because of the heterogeneity of breast cancer, limited biopsy tissue sometimes cannot represent the entire tumor, which will affect the treatment effect. Triple-negative breast cancer (TNBC) is a particular type of breast cancer defined by the absence of estrogen and progesterone receptor expression as well as the absence of ERBB2 amplification, which accounts for 15% to 20% of breast cancers [5]. It is more aggressive and has a low survival rate and lack of effective targeted therapy. If we can accurately distinguish triple-negative and nontriple-negative breast cancer, it will help our clinical decision-making.

Magnetic resonance imaging (MRI) is the most sensitive imaging technique for breast cancer detection. Recent studies have found that imaging omics models based on breast MRI have made a breakthrough in the differentiation of benign and malignant breast tumors [6, 7] and molecular subtypes [8, 9]. Radiomics is a noninvasive imaging technology that has great potential to evaluate the entire tumor features through the extraction of a large number of quantitative imaging features [10, 11]. In clinical practice, radiomics is expected to become an imaging biomarker for different tumors.

Precise segmentation of breast tumors as the mask is particularly important for radiomics model exploration and affects the efficacy of radiomics models. The current image segmentation methods include manual, semiautomatic, and fully automatic. Prior studies in the breast mainly used manual segmentation [8] or localizing region-based active contours algorithm [12] to determine the boundary of interest on images, which could be time and human consuming.

Deep learning is a kind of abstraction and simulation of the basic characteristics of the human brain or natural neural network. We have completed a preliminary study on the automatic segmentation of breast tumors in DCE-MRI images with deep learning models. The results show that this method has good repeatability and accuracy and can be used for automatic segmentation and measurement of breast tumors [13]. The purpose of this study was to investigate whether radiomics models based on automatic image segmentation of DCE-MRI can predict TNBC in a population of Chinese women.

## 2. Materials and Methods

This study was a retrospective study and was approved by the responsible institutional review board of Peking University First Hospital [IRB number: 2019(170)] with a waiver of informed consent.

### 2.1. Patients

**Figure 1:**
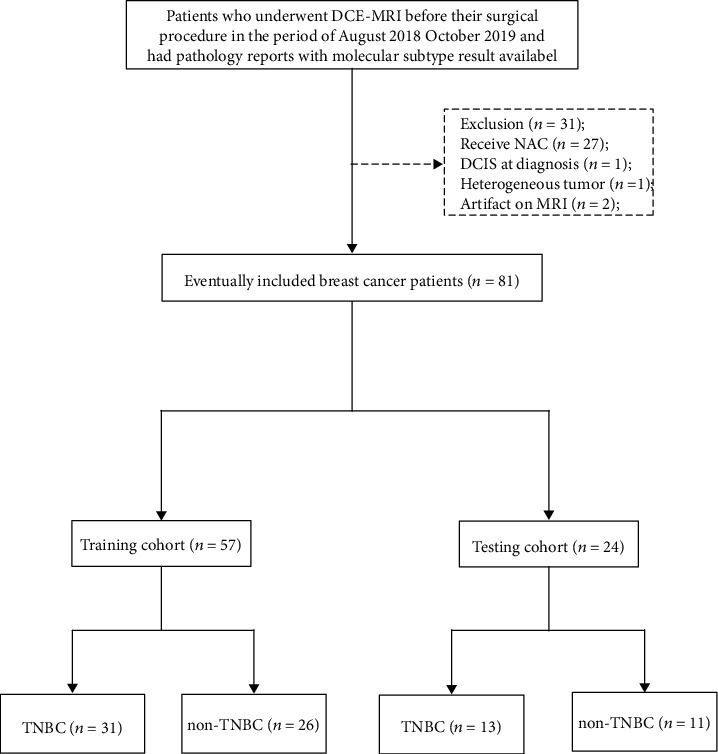
Flow chart of patient enrollment. (DCE-MRI: dynamic contrast-enhanced magnetic resonance imaging; NAC: neoadjuvant chemotherapy; DCIS: ductal carcinoma in situ; TNBC: triple-negative breast cancer; non-TNBC: nontriple-negative breast cancer.).

A consecutive group of patients who underwent DCE-MRI before surgery from August 2018 to October 2019 was queried. All the patients had molecular subtype results with surgery specimen pathology reports. The criteria for exclusion from the study were as follows: (I) has received any tumor-specific therapy before MRI exam include neoadjuvant chemotherapy (NAC), hormonal therapy, and radiotherapy; (II) stage 0 or ductal carcinoma in situ (DCIS) at diagnosis; (III) heterogeneous tumor which contains two or more molecular subtypes; (V) has artifacts on MRI exams. Finally, 81 women were recruited, with ages between 36 and 85 years. Among them, 4 women with multiple unilateral tumors were checked according to the pathological record, and the largest tumor mass was selected for analysis. The data were randomly divided into two datasets. Fifty-seven cases were randomly selected as the training cohort (TNBC = 31, non − TNBC = 26). The other 24 cases were left as the independent testing cohort (TNBC = 13, non − TNBC = 11). The patient enrollment process is depicted in Figure 1. Clinical characteristics of the patients are shown in Table 1. There was no significant difference in clinical characteristics between the training and testing cohorts.

### 2.2. MR Imaging

MR imaging was performed on a 1.5 T MRI system (Signa Twinspeed; GE Medical Systems, USA) with an eight-channel phased-array bilateral breast coil. The MRI protocol included axial T1-weighted imaging T2-weighted imaging, diffusion-weighted imaging, and DCE-MRI. Three-dimensional axial T1WI volume sequence of DCE MR imaging was performed every 58 s to scan 124 slices (TR 6 ms/TE 2.6 ms; FOV, 32 cm × 32 cm; matrix, 384 × 288; slice thickness, 2.4 mm; intersection gap, 0 mm; bandwidth, 62.5 Hz; and NEX,1). The DCE-MRI acquisitions were started after intravenous administration of 0.1 mmol/kg of Gd-DTPA (Magnevist, Bayer Schering Pharma, Germany), followed by a flush of 20 ml of saline solution with the flow of about 2 ml/s. The acquisition was repeated eight times, and each phase took 58 seconds.

### 2.3. Image Segmentation

The homemade deep learning segmentation model of breast tumor has been established and published [13]. The model runs on a hardware platform with GPU NVIDIA Tesla P100 16G, and the software includes Python 3.6, Pytorch 0.4.1, Opencv, Numpy, and Simple ITK.

**Figure 2:**
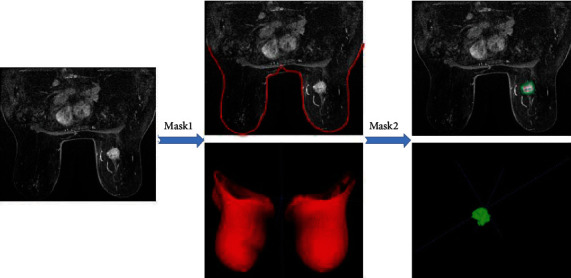
Example of Coarse-to-Fine segmentation of the deep learning segmentation model on DCE-MRI. (a) A DCE-MRI of a 57-year-old woman with TNBC on the third DCE phase. (b) Coarse segmentation (Mask 1, red) of bilateral breasts. (c) Fine segmentation (Mask 2, green) of the breast tumor. (DCE-MRI: dynamic contrast-enhanced magnetic resonance imaging; TNBC: triple-negative breast cancer.).

The segmentation model is 3D U-Net. The input is the images of the third postcontrast of DCE-MRI, and the output is the automatic segmentation of the tumor region. The breast tumor was segmented at the third postcontrast of DCE-MRI, to better distinguish it from background parenchyma. The algorithms use a Coarse-to-Fine segmentation method, first to segment the bilateral breast, and then segment the tumor lesion (Figure 2).

**Figure 3:**
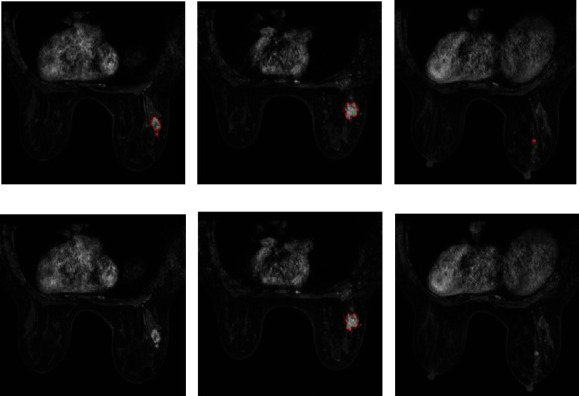
Example of manual revision of multiple tumors in the unilateral breast, only the largest tumor was selected. (a–c) Different slices including segmentation outlines by the deep learning segmentation model on DCE-MRI. (d–f) Manual selection of the largest tumor region. (DCE-MRI: dynamic contrast-enhanced magnetic resonance imaging.).

ITK-SNAP Toolbox v. 3.6.0 (http://www.itksnap.org/) was utilized for revising the automatically segmented tumor areas. Two dedicated breast radiologists (reader A and reader B, with and more than 17 years of experience in breast diagnosis, respectively) participated in the manual revision. The rules for manual revision are as follows: (1) labeling the tumor lesions with pathological record of molecular subtype; (2) if there are multiple tumors in the unilateral or bilateral breast, only the largest tumor was selected (Figure 3). After the revision, the overlay of the automatic segmentation tumor area and human annotation area was compared by Dice Similarity Coefficient (DSC).

### 2.4. Radiomics Feature Analysis and Modeling

The radiomics pipeline includes the following steps: (1) image preprocessing, (2) radiomics feature extraction, (3) radiomics model development, and (4) results inspection.

Images were preprocessed before extracting radiomics features and the pipeline of preprocessing was shown in Supplement Material S1. All the MRI images were filtered by Laplacian of Gaussian (LoG) filter, which was used to do image denoising and image edges detection. All the images were also performed wavelet transformation, which was used to do image denoising and improve the image quality. So there were three types of images, namely, “Original Images,” “LoG Images,” and “Wavelet Images.” All the images would be used for omics analysis.

The radiomics features were extracted utilizing the PyRadiomics software package in Python [14]. A total of 1,070 radiomics features were extracted from each ROI, containing 840 texture features, 216 first-order statistical features, and 14 shape-based features (Supplement Material S2 and Supplement Table S3).

In this research, the radiomics models were developed followed the below steps: data normalization (four methods), dimension reduction(two methods), feature selection(four methods, 20 features), and classifier (10 methods). A detailed list of available options can be found in Table 2. The 6400 (4 × 2 × 4 × 20 × 10) radiomics models were established through all possible combinations of all the methods. Cross-validation (CV) was performed with 5-fold on the training cohort to train and validate.

The statistical result presented includes the area under the curve (AUC), accuracy, sensitivity, specificity, and others for the training cohort and test cohort. Statistics of all models are sorted by the AUC on the testing cohort, hence, different models can be easily compared to find the best model. All the radiomics models were explored and tested on an open-source platform of Feature Explorer Pro (FAEPro, V 0.3.4) on Python (3.7.6) [15].

### 2.5. Statistical Analysis

Comparison of clinical characteristics between the training and testing cohorts was achieved by the Chi-square test or the Fisher's exact test using the SPSS 23.0 software package (SPSS, Inc., Chicago, IL, USA). Statistical significance was established at a *p* value < 0.05. The performance of the model was evaluated using receiver operating characteristic (ROC) curve analysis. The AUC was calculated for quantification. Graphpad Prism version 8 was used for analysis. The accuracy, sensitivity, and specificity were also calculated at a cutoff value that maximized the value of the Youden index.

## 3. Result

**Figure 4:**
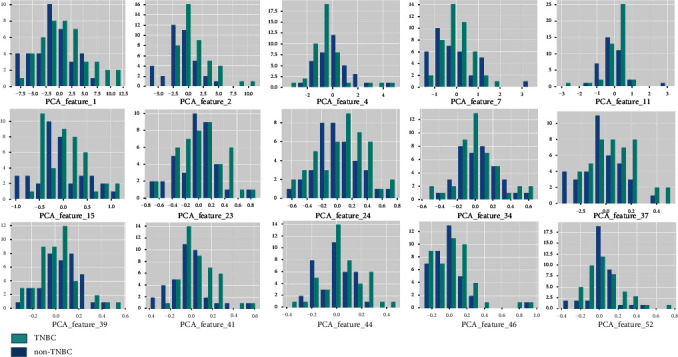
Histograms of selected features in TNBC and non-TNBC. (TNBC: triple-negative breast cancer; non-TNBC: nontriple-negative breast cancer; PCA: principal component analysis.).

**Figure 5:**
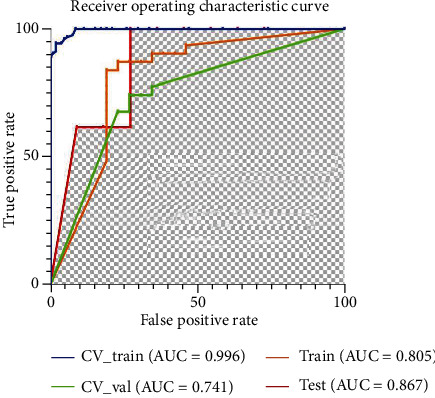
ROC curves of the radiomics model on different datasets. (ROC: receiver operating characteristic; AUC: area under the curve; cv: cross-validation; val: validation.).

The average DSC value of automatic segmentation for the tumor was 0.82. Finally, the chosen model using 15 features yielded the best performance in predicting TNBC or non-TNBC in the CV training (AUC = 0.996), CV validation (AUC = 0.741), training (AUC = 0.805), and testing (AUC = 0.867) cohorts. The pipeline of the radiomics model is listed in Table 3 and described in detail in Supplement Material S4. The selected 15 features for the model are shown in Table 4, and the histogram of each selected feature in TNBC and non-TNBC is shown in Figure 4. The statistical values of diagnosis in the testing cohort are shown in Table 5. The ROC curve is shown in Figure 5.

## 4. Discussion

DCE-MRI [16] is one of the main imaging methods for detecting breast cancer at present. Breast lesions are mainly diagnosed based on their morphologic and dynamic characteristics on dynamic contrast-enhanced (DCE) MRI. Radiomics in breast cancer has been applied for predicting molecular subtype, genomics, pathological complete response after NAC, residual cancer burden, and lymph node involvement [17–19]. Accurate segmentation is needed for quantitative features extraction, and manual segmentation by experienced radiologists is expected to be the “gold standard”, but it is very time-consuming and not suitable for large databases [20]. Various semiautomatic and automatic MRI segmentation methods have been developed [21, 22]. Nie et al. [21] reported a semiautomated tumor segmentation method that required the operator to indicate the beginning and ending slices containing the tumor and place an initial square-shaped ROI on one imaging slice. But the limitation of this study was that the square-shaped ROI may involve other body parts, which would affect the accuracy of the lesion segmentation. Lin et al. [22] pointed out that automated image segmentation provided a consistent criterion without the need for operator's help, but this might not work well when there were artifacts inside the chest wall muscle. Although the results of automated image segmentation are promising, errors due to blurred contrast and bias-field are common, and manual correction is often needed to ensure accuracy [23].

In this study, a deep-learning model for breast tumor segmentation was implemented. With our previous work, we had trained the 3D U-net model and used it in our clinical practice. We found that the deep learning segmentation is feasible and time-saving to perform fully automatic segmentation for the breast tumor on DCE-MRI images. After automatic segmentation of the tumor, its size and volume were automatically reported into a structured reporting system [13]. The automatic segmentation and reporting process could yield reasonable accuracy compared to the manual measurement process of radiologists. In order to evaluate the accuracy of the 3D mask of the automatic segmentation model, we compared the coverage of the predicted area and the manual segmentation through the DSC, which was as high as 0.82.

The results showed that the radiomics model based on automatic image segmentation could distinguish TNBC and non-TNBC with the AUC of 0.867 in the testing cohort. Wang et al. [23] had proved that the quantitative features of the breast tumor segmented at DCE-MRI using a semiautomated technique can predict triple-negative breast cancer with an AUC of 0.878. Our research has achieved similar performance, indicating that our method is feasible.

Our study has several limitations. First, it was a retrospective analysis of a small number of images from a single institution, and the MRI images with obvious artifacts were excluded. In some studies, it has been shown that Wiener or Median filters were very useful for removing the artifacts caused by the patient's respiration motion [24, 25]. In the future, we intend to use these filters to including images with artifacts for research and perform a large multicenter study to verify the feasibility of the radiomics model. Second, our study focused on the characterization of the tumor itself. Some studies had reported that analyses of the tumor and its surrounding parenchyma may improve the performance of subtype classification [14]. Third, Luminal A, Luminal B, and HER2-enriched patients were mixed together and compared with triple-negative patients in this study, but the heterogeneity among nontriple-negative breast cancer between the subtypes cannot be ignored. Thus, pairwise comparisons between molecular subtypes should be made in the future study.

## 5. Conclusion

In this paper, we used an automatic segmentation method based on deep learning to segment breast tumor regions on DCE-MRI. The radiomics model built by signatures extracted from the tumor region on the DCE-MRI performed well in distinguishing TNBC and non-TNBC. This showed that our method was not only time-saving but also effective. In the future, we intend to perform a multicenter study to verify the feasibility.

## Figures and Tables

**Table 1:** Clinical features of the patients.

Characteristic	Total	Training cohort	Test cohort	*P* value
Number (%)	81 (100)	57 (70.4)	24 (29.6)	
Age (year)^a^	52.5 ± 12.2	51.7 ± 12.4	53.7 ± 11.9	0.47
Molecular subtypes				0.98
TNBC	44	31 (70.5)	13 (29.5)	
Luminal A	13	9 (69.2)	4 (30.8)	
Luminal B	12	8 (66.7)	4 (32.3)	
HER2-enriched	12	9 (75.0)	3 (25.0)	

^a^Quantitative variables are expressed as mean ± standard deviation. (TNBC: triple-negative breast cancer; HER2: human epidermal growth factor receptor 2.).

**Table 2:** Accessible methods for all steps in the radiomics pipeline.

Radiomics pipeline	Method									
Data normalization	Min-max	Zscore	Mean	None						
Dimension reduction	PCA	PCC								
Feature selection	ANOVA	KW	RFE	Relief						
Classifier	SVM	LDA	MP	RF	LR	LASSO	AB	DT	GP	NB

(PCA: principal component analysis; PCC: Pearson correlation coefficient; ANOVA: analysis of variance; KW: Kruskal–Wallis test; RFE: recursive feature elimination; SVM: support vector machine; LDA: linear discriminant analysis; MP: multilayer perceptron, RF: recursive feature; LR: linear regression; LASSO: least absolute shrinkage and selection operator; AB: Adaboost; DT: decision tree; GP: Gaussian process; NB: naïve Bayes).

**Table 3:** The pipeline of the model with the best performance.

Modeling steps	Method
Data normalization	Min-max
Dimension reduction	PCA
Feature selection	KW
Classifier	SVM

(PCA: principal component analysis; KW: Kruskal–Wallis test; SVM: support vector machine).

**Table 4:** The selected features for the model according to validation performance.

Features	Coefficient in model
PCA_feature_1	0.932
PCA_feature_2	2.886
PCA_feature_4	-1.020
PCA_feature_7	0.329
PCA_feature_11	0.597
PCA_feature_15	1.014
PCA_feature_23	1.449
PCA_feature_24	0.980
PCA_feature_34	-1.338
PCA_feature_37	1.830
PCA_feature_39	-1.238
PCA_feature_41	1.412
PCA_feature_44	1.174
PCA_feature_46	0.932
PCA_feature_52	-0.897

(PCA: principal component analysis.).

**Table 5:** Performance of the radiomics model in the test data.

Statistics	Value
Accuracy	0.8333
AUC	0.8670
Sensitivity	0.9230
Specificity	0.7273

(AUC: area under the curve.).

## Data Availability

The data of clinical information and radiomics results used to support the findings of this study are included within the article. The data of the radiomics pipeline used to support the findings of this study are included within the supplementary material.

## Supplementary Materials

Supplementary Materials Supplementary material related to this article can be found in the online version. Supplement Material S1 shows the pipeline of images preprocessing. Supplement Material S2 and S3 show the pipeline of radiomics feature extraction and the radiomics features used in this study. Supplement Material S4 shows the details of the radiomics modeling pipeline.
