# Supplementary material for: Radiomics Analysis Based on Automatic Image Segmentation of DCE-MRI for Predicting Triple-Negative and Nontriple-Negative Breast Cancer
Source: Comput Math Methods Med. 2021 Aug 10;2021:2140465. doi: 10.1155/2021/2140465 (PMC8371618; doi:10.1155/2021/2140465)
Supplement: Supplementary Materials — Supplementary material related to this article can be found in the online version. Supplement Material S1 shows the pipeline of images preprocessing. Supplement Material S2 and S3 show the pipeline of radiomics feature extraction and the radiomics features used in this study. Supplement Material S4 shows the details of the radiomics modeling pipeline. [file 2140465.f1.docx]

**S1:** **The pipeline of the images pre-processing**

**Normalization:**

MR signal is usually relative, with large differences between scanners and vendors. By normalizing the image before feature calculation, this confounding effect may be reduced. However, if only one specific scanner is used, or the images reflect some absolute world value (e.g. ADC maps, T2 maps (NOT T2 weighted)), consider disabling the normalization. Normalize Scale: 100 (This allows you to use more or less the same bin width). Bin Width: 5.

**First order specific settings:**

When normalizing, gray values below the mean will be negative. Shifting by 300 (3 StdDevs * 100) ensures that the majority of voxels is positive (only outliers >3 SD lower than the mean will be negative). Voxel Array Shift: 300.

**Image types to use:** "Original" for unfiltered images. “LoG”: The Laplacian of Gaussian (LoG) band pass filter was applied to the input original image for fine to coarse texture (filter width: fine, σ=1.0; medium, σ=3.0; coarse, σ=5.0). If the in-plane spacing is large (> 2mm), consider removing sigma value 1. “Wavelet”: We performed a three-dimensional wavelet transformation on the images. The sym8 function was selected to perform multiscale wavelet decomposition. The decomposed high-frequency coefficients were enhanced, and the wavelet reconstruction was carried out depending on the high- and low-frequency coefficients to obtain the enhanced images. So resulting in 8 frequency domain data: LLH, LHL, HLL, LHH, HHL, HLH, HHH, LLL.

**S2: Radiomics feature extraction**

To extract the radiomics features, the following 3 steps related to the parameter setting were performed (1):

1. Three settings are specified as follows: “bin Width”, “label”, and “voxel Array Shift”.

2. Three image types are enabled (“Original”, “LoG” (Laplacian of Gaussian) and “Wavelet”), with custom settings specified for “LoG” (“sigma: [1.0, 3.0, 5.0]”) and “Wavelet” (“[LLH, LHL, HLL, LHH, HHL, HLH, HHH, LLL]”).

3. The following six feature classes are defined: “shape”, “first order”, “glcm”, “glrlm”, “glszm” and “gldm”; the classes are enabled with all possible features in the respective class.

The following three groups of radiomics features were used (2): first-order statistical features (n=18), shape-based features (n=14), and textural features (n=24 GLCM +16 GLRLM +16 GLSZM +14 GLDM) (Table S2).

Therefore, 216 ((1 Original + 3 LoG + 8 Wavelet) ×18) first-order statistical features, 14 shape-based features and 840 ((1 Original + 3 LoG + 8 Wavelet) × (24+16+16+14)) texture features (for a total of 1070 (216+14+840) radiomics features) were extracted from each volume of interest (VOI).

**S3: Radiomics features used in this study.**

|  |  | Features | |
| --- | --- | --- | --- |
| **First-order Statistical Features** |  | 10^th^ Percentile | |
| **(n=18)** |  | 90^th^ Percentile | |
|  |  | Energy | |
|  |  | Entropy | |
|  |  | Interquartile Range | |
|  |  | Kurtosis | |
|  |  | Maximum | |
|  |  | Mean Absolute Deviation | |
|  |  | Mean | |
|  |  | Median | |
|  |  | Minimum | |
|  |  | Range | |
|  |  | Robust Mean Absolute Deviation | |
|  |  | Root Mean Squared | |
|  |  | Skewness | |
|  |  | Total Energy | |
|  |  | Uniformity | |
|  |  | Variance | |
| **Shape Features** |  | Elongation |  |
| **(n=14)** |  | Flatness | |
|  |  | Least Axis Length | |
|  |  | Major Axis Length | |
|  |  | Maximum 2D Diameter (Column) | |
|  |  | Maximum 2D Diameter (Row) | |
|  |  | Maximum 2D Diameter (Slice) | |
|  |  | Maximum 3D Diameter | |
|  |  | Mesh Volume | |
|  |  | Minor Axis Length | |
|  |  | Sphericity | |
|  |  | Surface Area | |
|  |  | Surface Volume Ratio | |
|  |  | Voxel Volume | |
| **Textural Features: Gray Level Co-occurrence Matrix (GLCM) Features** |  | Autocorrelation | |
| **(n=24)** |  | Cluster Prominence | |
|  |  | Cluster Shade | |
|  |  | Cluster Tendency | |
|  |  | Contrast | |
|  |  | Correlation | |
|  |  | Difference Average | |
|  |  | Difference Entropy | |
|  |  | Difference Variance | |
|  |  | Inverse Difference (ID) | |
|  |  | Inverse Difference Moment (IDM) | |
|  |  | Inverse Difference Moment Normalized (IDMN) | |
|  |  | Inverse Difference Normalized (IDN) | |
|  |  | Informational Measure of Correlation (IMC) 1 | |
|  |  | Informational Measure of Correlation (IMC) 2 | |
|  |  | Inverse Variance | |
|  |  | Joint Average | |
|  |  | Joint Energy | |
|  |  | Joint Entropy | |
|  |  | Maximal Correlation Coefficient (MCC) | |
|  |  | Maximum Probability | |
|  |  | Sum Average | |
|  |  | Sum Entropy | |
|  |  | Sum of Squares | |
| **Textural Features: Gray Level Run Length Matrix (****GLRLM) Features** |  | Gray Level Non-Uniformity (GLN) | |
| **(n=16)** |  | Gray Level Non-Uniformity Normalized (GLNN) | |
|  |  | Gray Level Variance (GLV) | |
|  |  | High Gray Level Run Emphasis (HGLRE) | |
|  |  | Long Run Emphasis (LRE) | |
|  |  | Long Run High Gray Level Emphasis (LRHGLE) | |
|  |  | Long Run Low Gray Level Emphasis (LRLGLE) | |
|  |  | Low Gray Level Run Emphasis (LGLRE) | |
|  |  | Run Entropy (RE) | |
|  |  | Run Length Non-Uniformity (RLN) | |
|  |  | Run Length Non-Uniformity Normalized (RLNN) | |
|  |  | Run Percentage (RP) | |
|  |  | Run Variance (RV) | |
|  |  | Short Run Emphasis (SRE) | |
|  |  | Short Run High Gray Level Emphasis (SRHGLE) | |
|  |  | Short Run Low Gray Level Emphasis (SRLGLE) | |
| **Textural Features: Gray Level Size Zone Matrix (****GLSZM) Features** |  | Gray Level Non-Uniformity (GLN) | |
| **(n=16)** |  | Gray Level Non-Uniformity Normalized (GLNN) | |
|  |  | Gray Level Variance (GLV) | |
|  |  | High Gray Level Zone Emphasis (HGLZE) | |
|  |  | Large Area Emphasis (LAE) | |
|  |  | Large Area High Gray Level Emphasis (LAHGLE) | |
|  |  | Large Area Low Gray Level Emphasis (LALGLE) | |
|  |  | Low Gray Level Zone Emphasis (LGLZE) | |
|  |  | Size-Zone Non-Uniformity (SZN) | |
|  |  | Size-Zone Non-Uniformity Normalized (SZNN) | |
|  |  | Small Area Emphasis (SAE) | |
|  |  | Small Area High Gray Level Emphasis (SAHGLE) | |
|  |  | Small Area Low Gray Level Emphasis (SALGLE) | |
|  |  | Zone Entropy (ZE) | |
|  |  | Zone Percentage (ZP) | |
|  |  | Zone Variance (ZV) | |
| **Textural Features: Gray Level Dependence Matrix (****GLDM) Features** |  | Dependence Entropy (DE) | |
| **(N=14)** |  | Dependence Non-Uniformity (DN) | |
|  |  | Dependence Non-Uniformity Normalized (DNN) | |
|  |  | Dependence Variance (DV) | |
|  |  | Gray Level Non-Uniformity (GLN) | |
|  |  | Gray Level Variance (GLV) | |
|  |  | High Gray Level Emphasis (HGLE) | |
|  |  | Large Dependence Emphasis (LDE) | |
|  |  | Large Dependence High Gray Level Emphasis (LDHGLE) | |
|  |  | Large Dependence Low Gray Level Emphasis (LDLGLE) | |
|  |  | Low Gray Level Emphasis (LGLE) | |
|  |  | Small Dependence Emphasis (SDE) | |
|  |  | Small Dependence High Gray Level Emphasis(SDHGLE) | |
|  |  | Small Dependence Low Gray Level Emphasis (SDLGLE) | |

**S4:** **Details of the radiomics modeling pipeline**

*Normalization:* *Min-Max*

Min-Max normalization was used to obtain data with the same ranges. For each feature vector, the L2 norm was calculated. Additionally, the feature values were divided by the L2 norm. Then, the feature vector was mapped to a unit vector, and the feature values were distributed in an equal range with equal means and standard deviations.

*Dimension reduction: PCA*

Since the dimension of the feature space was high, a principal component analysis (PCA) of the feature matrix was performed to reduce the data dimensionality. During this process, by linear combinations, the original features were reidentified as new variables, i.e., principal components (3). Then, the feature vector of the transformed feature matrix was independent of each other.

*Feature selection: KW*

The Kruskal-Wallis test was used to select the features before building the model. The Kruskal-Wallis test is a common method used to explore the significant features corresponding to the labels, and all features had corresponding *P*-values. If the features had *P*-values smaller than 5%, the features were significant for the corresponding label, had value for further analysis, and were reserved.

*Classifier: SVM*

Support vector machine (SVM) is an effective and robust classifier used to build the model. The kernel function has the ability to map the features into a higher dimension to search the hyper-plane to separate the cases with different labels. Here, we used the linear kernel function because it is easier to explain the coefficients of the features in the final model. Hinge loss (soft-margin) was used as a loss function in the support vector classification, with the following equation: $L(y_{i},f(x_{i}))=max(0,1-y_{i}f(x_{i}))$.

**References**

1. Pyradiomics community. Customizing the extraction. 2016. Avaliable from: https://pyradiomics.readthedocs.io/en/latest/customization.html. [Accessed Jan 10,2020].

2. Pyradiomics community. Radiomics feature extraction in Python. 2016. Avaliable from: https://github.com/radiomics/pyradiomics. [Accessed Oct 26,2020].

3. Fangying Chen XM, Shuai Li, Zhihui Li, Yan Jia, Yuwei Xia, Minjie Wang, Fu Shen, Jianping Lu. MRI-based radiomics of rectal cancer: assessment of the local recurrence at the site of anastomosis. Acad Radiol 2020;S1076-6332(20):30567-30565.
